# Current Evidence and Surgical Strategies in the Management of Greater Tuberosity Fracture–Dislocations: A Narrative Review

**DOI:** 10.3390/jcm14145159

**Published:** 2025-07-21

**Authors:** Gabriele Colò, Federico Fusini, Luca Faoro, Giacomo Popolizio, Sergio Ferraro, Giorgio Ippolito, Massimiliano Leigheb, Michele Francesco Surace

**Affiliations:** 1 Department of Orthopaedics and Traumatology, Sant’Anna Hospital, Via Ravona 20, 22042 Como, Italy; 2 Multidisciplinary Research Center for Pathology and Surgery of the Musculoskeletal System, Department of Biotechnology and Life Sciences (DBSV), Insubria University, 21100 Varese, Italy; 3 Department of Orthopaedic and Traumatology, Orthopaedic and Trauma Centre, University of Turin, Via Zuretti 29, 10124 Turin, Italy; 4 Department of Surgery, Orthopedics and Traumatology, Asl1 SSR Liguria, 18039 Sanremo, Italy; 5 Trauma & Orthopaedic Department, Icot Hospital “Marco Pasquali”, 04100 Latina, Italy; 6 Orthopaedics and Traumatology Unit, San Gaudenzio Clinic (Monza Polyclinic Group), Via Enrico Bottini 3, 28100 Novara, Italy

**Keywords:** arthroscopic fixation, greater tuberosity fracture, open fixation, proximal humerus, shoulder pain

## Abstract

**Background**: Greater tuberosity fracture–dislocations (GTFDs) represent a distinct subset of proximal humerus fractures, occurring in up to 57% of anterior glenohumeral dislocations. Malreduction may result in impingement, instability, and functional limitation. Treatment is influenced by the displacement magnitude and direction, bone quality, and patient activity level. **Methods**: This narrative review was based on a comprehensive search of PubMed, Scopus, and Web of Science for English-language articles published between January 2000 and March 2025. Studies on pathomechanics, classification, diagnosis, treatment, and outcomes of GTFDs in adult and pediatric populations were included. Data were analyzed to summarize the current evidence and identify clinical trends. **Results**: A displacement ≥ 5 mm is the standard surgical threshold, though superior or posterosuperior displacement ≥ 3 mm—and ≥2 mm in overhead athletes—may justify surgery. Conservative treatment remains appropriate for minimally displaced fractures but is associated with up to 48% subacromial impingement and 11% delayed surgery. Surgical options include arthroscopic repair for small or comminuted fragments and open reduction and internal fixation (ORIF) with screws or plates for larger, split-type fractures. Locking plates and double-row suture constructs demonstrate superior biomechanical performance compared with transosseous sutures. Reverse shoulder arthroplasty (RSA) is reserved for elderly patients with poor bone stock, cuff insufficiency, or severe comminution. Pediatric cases require physeal-sparing strategies. **Conclusions**: GTFDs management demands an individualized approach based on fragment displacement and direction, patient age and activity level, and bone quality. While 5 mm remains the common threshold, lower cutoffs are increasingly adopted in active patients. A tiered treatment algorithm integrating displacement thresholds, fracture morphology, and patient factors is proposed to support surgical decision making. The incorporation of fracture morphologic classifications further refines fixation strategy. Further prospective and pediatric-specific studies are needed to refine treatment algorithms and validate outcomes.

## 1. Introduction

Greater tuberosity fractures (GTFs) comprise approximately 1–14% of all proximal humerus fractures [1]; of these injuries, 5–57% occur in association with an anterior glenohumeral dislocation, whereas 7–30% of anterior dislocations involve a GT fracture [2,3,4]. GTF dislocations (GTFDs) often result from direct trauma to the acromion or glenoid during dislocation or from avulsion and shearing mechanisms. They typically involve the rotator cuff footprint, which can significantly affect shoulder function and stability. Despite the clinical relevance of these injuries, no standardized management algorithm exists to date. Given the increasing incidence of shoulder trauma across both older and younger populations, understanding the management of GTFDs is essential for clinicians [5].

Management strategies range from conservative approaches for nondisplaced fractures to surgical fixation in displaced cases. However, the threshold for surgical intervention remains variably defined across studies [6]. Recent advancements in surgical techniques, including arthroscopic fixation and earlier open reduction methods, have expanded options for functional restoration [7]. Nevertheless, the clinical superiority of one approach over another remains unclear due to a lack of high-quality comparative studies [8].

Moreover, the literature indicates a growing emphasis on individualized therapeutic strategies that consider patient age, functional demand, and overall medical condition [9,10,11,12].

This narrative review synthesizes the current literature on GTFDs, with a focus on pathophysiology, diagnostic challenges, and management strategies. It aims to consolidate key clinical insights and identify areas where further research is needed to inform future treatment protocols.

## 2. Search Strategy

This narrative review was conducted through a comprehensive literature search using PubMed/MEDLINE, Scopus, and Web of Science databases for English-language publications from January 2000 to March 2025. The search strategy incorporated combinations of the following keywords and MeSH terms: “greater tuberosity fracture,” “proximal humerus fracture,” “rotator cuff avulsion,” “shoulder trauma,” “nonunion,” “arthroscopic fixation,” “displacement threshold,” and “functional outcomes.”

We included original research articles, clinical trials, retrospective series, case reports with clinical relevance, systematic reviews, and selected biomechanical studies. Article selection was based on clinical relevance, methodological quality, and the specific focus on displacement thresholds, diagnostic criteria, treatment strategies, or outcomes for GTFDs in adult or pediatric populations. Although no formal PRISMA criteria were applied, studies were excluded if they lacked focus on GTFDs, were not peer-reviewed, or were not available in English.

Preference was given to studies with larger cohorts, longer follow-up, or higher evidence to enhance clinical relevance. This review protocol was not prospectively registered. No formal risk of bias or quality assessment tools were applied, in keeping with the narrative nature of the study. 

## 3. Mechanism of Injury

Various mechanisms have been described for GTFDs, though their pathobiomechanics remain incompletely understood [1,13,14,15]. Traditionally, these injuries have been attributed to avulsion forces generated by a sudden and forceful contraction of the rotator cuff during shoulder abduction and external rotation. However, not all displacement patterns support this model, suggesting that additional mechanisms may be involved. Other proposed mechanisms include direct trauma to the lateral aspect of the shoulder, falls onto an outstretched hand (with the elbow extended or flexed), and seizure-related convulsions [16].

In a retrospective study of 103 patients, Bahrs et al. reported that 47.6% of GTFDs resulted from direct trauma, while 32% were due to indirect mechanisms; 20.4% of patients could not recall the exact mechanism due to concurrent neurological impairment. Interestingly, in cases associated with anterior shoulder dislocation, the authors observed that avulsion was unlikely to be the primary cause: 78% of fractures showed either no displacement or inferior displacement, contradicting the classic superior–posterior avulsion model [1]. This supports a multifactorial origin, with implications for preoperative planning and fixation strategy [13,14,15].

These findings support alternative theories, such as shearing of the GT against the glenoid rim during dislocation or impaction of the tuberosity against the acromion in forced elevation. Furthermore, inferior displacement patterns—identified in up to 25% of cases—suggest compressive rather than tensile loading [17].

Recent investigations [18] have reported that in 20% to 32% of glenohumeral dislocations, the GT exhibits inferior displacement. These patterns may reflect hyperabduction-related mechanisms in which the GT impacts the undersurface of the lateral acromion, functioning as a fulcrum that facilitates anterior humeral head dislocation. Alternatively, they may result from direct compressive forces driving the tuberosity against the inferior aspect of the glenoid [19].

Bone quality also plays a pivotal role. Osteoporosis and compromised trabecular architecture, especially in the elderly, may predispose to GT failure under relatively low-energy conditions. Instead, high-energy traumas (for example, car accidents or sports injuries) are predominant in younger subjects [20,21,22].

Overall, the mechanism of injury in GTFDs is likely multifactorial, involving a complex interplay of rotator cuff dynamics, joint position at the time of trauma, external impact vectors, and patient-specific skeletal factors. Each factor may inform both classification and treatment planning [23,24,25,26].

## 4. Classification

In 1970, Neer [27] introduced the most commonly adopted system for categorizing proximal humeral fractures, segmenting the proximal humerus into the GT, lesser tuberosity, humeral head, and shaft, and referring to these fracture components as “parts”. The objective was to distinguish fracture components as either stable or unstable, with displacement originally defined as greater than 1 cm; however, the current consensus considers GT displacement significant when exceeding 5 mm [28,29] (Table 1).

The AO/OTA classification system presents greater complexity compared with Neer’s and relies on the fracture location, the presence of articular surface involvement, and features such as metaphyseal impaction, angular deformity, or fragmentation [30].

However, both the Neer and AO/OTA classifications show limited interobserver reliability and clinical applicability when used to assess isolated GTFs. In particular, the identification of displacement greater than 5 mm yields kappa values as low as 0.31–0.35 on radiographs [31,32,33,34,35], underscoring the variability in interpretation and the need for more morphology-based approaches.

**Figure 1:**
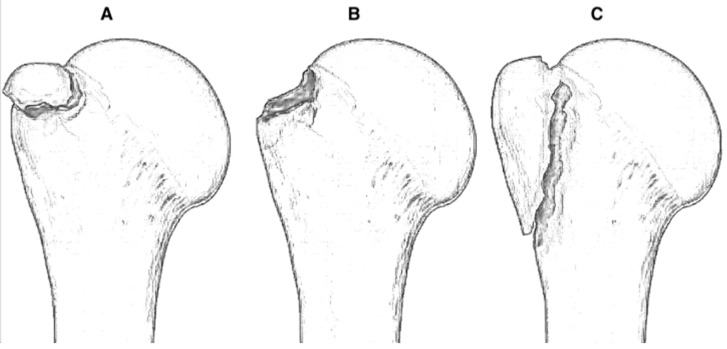
Illustration of the three types described by Mutch et al.: (**A**) avulsion type, (**B**) depression type, and (**C**) split type. Each subtype is associated with specific displacement patterns and fixation strategies.

Given these limitations in reliability and clinical applicability, there has been a progressive shift toward characterizing the morphological features of the GTF fragment, which may influence the choice of fixation method. Mutch et al. proposed a classification of GTFs into three types: avulsion, split, and depression fractures (Figure 1). The first type involves a small fragment of bone and represents a bony avulsion of the rotator cuff footprint. Split-type fractures are characterized by a sizable bone fragment with a vertical fracture trajectory extending from the rotator cuff footprint junction to the surgical neck level. Depression-type fractures resemble a Hill–Sachs lesion but are situated more laterally on the GT rather than on the humeral head articular surface [18].

In a recent literature review involving 55 GTFs, the avulsion, split, and depression types accounted for 29%, 36%, and 35%, respectively. Depression-type injuries demonstrated minimal displacement and required operative management in only 7% of instances. In contrast, avulsion and split configurations exhibited displacement exceeding 5 mm in roughly 35% and 38% of cases, respectively. The application of this classification scheme has meaningful consequences in selecting the appropriate surgical fixation strategy [17].

## 5. Clinical Examination

The main clinical finding is pain combined with visible or palpable swelling localized over the lateral aspect of the shoulder. Painfulness is exacerbated by movement, particularly abduction and external rotation, as these movements involve the rotator cuff muscles attached to the GT.

Reduced active and passive ROM, especially in abduction, occurs due to involvement of the supraspinatus, and external rotation, occur due to involvement of the infraspinatus and teres minor [36,37,38,39,40].

A pronounced subacromial groove or large shoulder deformity may raise suspicion of humeral head dislocation. Ecchymosis around the shoulder may be present. Concomitant injuries of the ipsilateral elbow and wrist may be present and should be examined [17].

A careful neurologic examination should be performed before and after any reduction; neurologic deficits are present in approximately 5.7% to 34% of cases, and axillary nerve injury is the most common deficit and occurs in 63% of cases. In 21% of cases, brachial plexus injury is involved [2].

Vascular complications are uncommon but become more prevalent in the presence of iatrogenic fractures of the surgical neck, in individuals over 50 years of age, with axillary artery atherosclerotic changes, and when neurological impairments are simultaneously observed [41,42,43].

However, clinical evaluation may be confounded by soft tissue swelling, pain inhibition, or coexisting injuries, which can obscure subtle signs of GTFs. In cases with apparent reduction of dislocation but persistent dysfunction, a missed or underestimated GT displacement should be suspected and further imaging considered [36,38].

## 6. Radiographic Evaluation

It is essential that radiographs include an anteroposterior (AP) view, scapular plane (Grashey view), scapular Y, and axillary lateral view [44,45]. Assessment of the radiographic images should be primarily aimed at detecting occult fractures of the humeral neck. Shaw et al. identified hidden humeral neck fractures in 7.4% of GT injuries [46], particularly in cases exhibiting inferior displacement of the GT [47]. In the context of a fracture–dislocation, up to 26% of reductions may result in iatrogenic humeral neck fractures, which carry significant clinical implications, as outcomes may be adversely affected [48,49]. Contributing factors include age above 50 years, female sex, and GT size exceeding 40% of the span between the fracture site and the medial calcar. When an occult fracture is suspected in patients older than 50 years, a Computed Tomography (CT) scan should be performed before proceeding with reduction [50].

In the presence of associated dislocation, prompt reduction of the shoulder is warranted. Patients should be counseled about the potential risk of an iatrogenic fracture during the reduction attempt. If the patient is under 50 years of age, has adequate bone density, and lacks radiographic signs suggestive of a neck fracture, a closed reduction under moderate sedation in the emergency department is acceptable, but it is advisable to limit attempts to a single trial [17]. Otherwise, closed reduction should be carried out in the operating theater, where general anesthesia and full neuromuscular relaxation can be achieved. Optimal muscle relaxation helps lower the likelihood of an iatrogenic fracture [49].

If the closed reduction is successful, shoulder immobilization with an abduction brace positioned in slight abduction can be applied to reduce the extent of fragment displacement. If closed reduction fails, open reduction should be undertaken without delay to improve patient outcomes and reduce the incidence of avascular necrosis [51], which may reach 19% if reduction is postponed beyond 24 h [52]; in one patient series, postponing both reduction and surgical fixation beyond 48 h resulted in avascular necrosis in all individuals [51,52].

Once open reduction is performed, even minimal residual displacement may progress, as the GT fragment undergoes secondary displacement in up to 51% of cases [53]. Therefore, fixation is generally preferred to reduce the risk of requiring additional procedures [49].

Following glenohumeral reduction, the GT may remain displaced by more than 1 cm in 25–49% of cases [2], and exceed 5 mm in 38% of instances [53]. However, discrepancies can occur due to differences in radiographic views and the methodologies employed to assess displacement. It is recommended to assess the degree of displacement using the AP view in external rotation along with the axillary lateral and/or scapular Y projections [54,55]. This standardized radiographic positioning minimizes misinterpretation of fragment displacement and should be emphasized in all suspected GTFD cases.

CT imaging, with or without 3D reconstructions, may offer supplementary insights into fracture configuration, although certain studies have demonstrated only a slight improvement—approximately 5%—in the surgeon’s decision making confidence [56].

Magnetic Resonance Imaging (MRI) does not appear to assist in surgical decision making [57], but it may show rotator cuff tears occurring in 38–100%, labral tears in 50–56%, and biceps tendon injuries in 41% of patients [58]. A stepwise approach is recommended:

(1) Begin with standard radiographs (AP in external rotation, scapular Y, axillary lateral);

(2) If clinical suspicion persists or inferior displacement is present, obtain CT to rule out occult neck fractures, especially in patients > 50 years old;

(3) Reserve MRI for persistent pain beyond 3 months or suspected soft tissue pathology.

Many authors advise against concurrent repair of the fracture and capsulolabral complex because it can significantly increase stiffness. In cases where pain persists beyond 3 months or weakness or instability is present, an MRI may be helpful to assess soft tissue damage [17].

## 7. Management

### 7.1. Non-Operative Treatment

#### 7.1.1. Indications

The displacement of the GT fragment remains the primary radiographic criterion for determining suitability for non-operative treatment. Historically, a threshold of 5 mm has been widely accepted as the upper limit for conservative management [28]. However, increasing attention has been paid to the biomechanical implications of even small displacements.

Several recent studies have demonstrated that minimal displacements, particularly those exceeding 3 mm, can significantly impair rotator cuff function and clinical outcomes, especially in younger and more active individuals [59]. Moreover, posterosuperior displacement has been specifically associated with poorer functional results when compared with displacement in other directions [60].

While a 5 mm displacement threshold is generally appropriate for the sedentary population, a stricter threshold of 3 mm has been advocated for young adults, athletes, and individuals engaged in overhead or physically demanding activities [61,62]. In addition, superior displacement as small as 2 mm has been associated with subacromial impingement and disruption of rotator cuff biomechanics, further justifying early surgical intervention in selected cases [63,64].

While current thresholds are supported by biomechanical and observational data, evidence remains limited to small cohorts and expert opinion. A recent case showed that subacromial impingement may develop despite minimal displacement, reinforcing the need for individualized risk–benefit assessment in young, active patients [23].

#### 7.1.2. Rehabilitation Protocols

Physiotherapy plays a central role in the recovery of shoulder function, not only in the context of GTFDs but also in related shoulder pathologies [65,66,67,68]. Hodgson et al. demonstrated that early mobilization in proximal humerus fractures leads to faster recovery, reduced pain, and no increase in complication rates, supporting a more proactive rehabilitation strategy in appropriate cases. Accordingly, some clinicians advocate for initiating passive and even active assisted range of motion (ROM) exercises within the first few weeks after injury in patients with stable fracture alignment [69].

Conversely, other authors recommend a more conservative protocol to minimize the risk of secondary displacement. This typically involves immobilization in a shoulder brace with an abduction pillow for six weeks. X-rays are performed on a weekly basis during the initial three weeks to monitor for secondary displacement. Passive mobility exercises are initiated after the third week, followed by active motion at six weeks and muscle strengthening at twelve weeks [17].

Adjunctive measures such as transcutaneous electrical nerve stimulation (TENS) and ice application may assist in acute pain management, especially in the early phase or after exercise sessions [70]. Corticosteroid injections combined with local anesthetics have also been proposed in cases of persistent inflammation and restricted ROM. These interventions, when administered within the first eight weeks post-injury, are supported by high-level evidence indicating reduced pain and improved shoulder mobility [71,72,73].

In the absence of significant structural damage, conservative management for three to six months is often the initial treatment of choice. The rehabilitation program typically progresses through three phases: acute pain control, restoration of passive and active ROM, and eventual strengthening. During the early recovery period, patients should avoid overhead activities to protect the healing rotator cuff and minimize stress on the tuberosity fragment.

#### 7.1.3. Outcomes

Functional outcomes following conservative treatment of minimally displaced GTFDs appear favorable in well-selected patients. In a cohort of 37 individuals exhibiting displacement below 5 mm post-reduction, Dussing et al. documented an average anterior elevation of 145° and a mean Constant score of 75 at a 4.9-year follow-up. None of the patients underwent revision surgery for subsequent displacement, though three presented with recurrent instability [33].

In another study involving 27 patients, a similar mean Constant score of 83 was achieved. However, 48% of patients developed subacromial impingement, and 11% eventually required surgical fixation [74].

For patients who do not respond to conservative treatment, delayed surgical intervention may yield excellent results. Kim et al. reported on nine patients with minimally displaced GTFs and persistent shoulder pain eight months after injury. All patients exhibited partial-thickness rotator cuff tears, and three presented with Bankart lesions. Arthroscopic subacromial decompression, rotator cuff debridement, and Bankart repair led to excellent clinical outcomes with no recurrent instability at a mean follow-up of 2.4 years [75].

### 7.2. Surgical Treatment

#### 7.2.1. Indications

Methods employed for managing GTFDs can encompass both open and arthroscopic techniques. The arthroscopic method is minimally invasive and offers the additional advantage of assessing and treating intra-articular injuries. The choice between open and arthroscopic intervention is primarily influenced by the configuration of the fracture, the dimension of the fragment, the quality of the bone, and the surgeon’s expertise and inclination [76,77], while reverse shoulder arthroplasty (RSA) may be considered in elderly patients with poor bone quality or severe comminution [22,38,39]. The current literature suggests that arthroscopic repair is appropriate when the fracture is <2 cm displaced, does not extend beyond the surgical neck, and the surgery occurs within two weeks of injury [78].

In a recent systematic review analyzing 16 studies, a 5 mm displacement threshold was used as the surgical indication in 68.7% of cases. Only one study used a 3 mm threshold, and another used 10 mm. The mean time from injury to surgery (across 13 studies and 310 shoulders) was 10.12 days (range: 1.3–38.9 days) [10].

Open or mini-open transosseous repair using figure-of-eight nonabsorbable sutures produced excellent outcomes in 73.5% and very good outcomes in 17.6% of patients [79]. Nevertheless, biomechanical investigations have shown that this method exhibits the lowest resistance to failure at 5 mm displacement when compared with alternative techniques, such as double-row suture bridge, locking plate stabilization, and cancellous screw [80,81].

#### 7.2.2. Fixation Techniques

Arthroscopic fixation is generally preferred in instances of minor or multifragmentary fractures, including avulsion-related lesions. A dual-row suture bridge arrangement in the beach-chair position is commonly employed [82].

After routine diagnostic arthroscopy, the subacromial bursa and fracture-associated hematoma are excised, and the fracture site is readied. Reduction is achieved arthroscopically using a grasper; if this fails, conversion to open reduction remains an option. Cancellous screws can be inserted through a mini-open or percutaneous incision to achieve interfragmentary compression. Both direct arthroscopic-assisted and indirect fluoroscopic reduction techniques have been described [33,83].

Biomechanical data show that cancellous screws have greater yield strength than transosseous sutures, although plate fixation and double-row suture anchor constructs surpass both [24,84]. Use of a cerclage wire to reinforce the screw construct further increases fixation strength, potentially matching that of suture anchor methods [85].

Nonetheless, screw fixation may be unsuitable for comminuted or small fragments due to the risk of further fragmentation. This technique is more appropriate in younger male patients with large split fractures and good bone quality [33]. Interfragmentary screws offer high primary stability but have been linked with higher reoperation rates, especially in osteoporotic bone [25].

For extensive split-type fractures that propagate distally past the surgical neck, an alternative involves passing nonabsorbable sutures through the rotator cuff and securing them to a pre-shaped humeral plate. This plate should be positioned 5 mm distal to the most superior GT edge to avoid subacromial impingement. Drawbacks include increased surgical exposure through the deltopectoral approach and higher cost, although newer low-profile plates specifically designed for GTFs are now available. Postoperatively, patients may be immobilized in slight abduction to reduce tension on the repair construct. Passive mobilization is typically allowed after one week to mitigate stiffness. Active ROM begins at six weeks, followed by strengthening exercises at twelve weeks [17].

While locking plates and double-row anchors offer strong biomechanical fixation, each technique has limitations [82,83]. Potential issues such as anchor pull-out, screw loosening, and soft tissue irritation should be considered, especially in osteoporotic or comminuted fractures [84,85]. Surgical choices should balance mechanical strength with patient-specific risk factors.

#### 7.2.3. Outcomes

Despite promising clinical outcomes, most available studies fail to isolate GTFDs from general GTF populations, limiting the generalizability of findings. Dimakopoulos et al. reported excellent or very good outcomes in 91% of patients treated surgically for GTFDs, with all returning to work at full capacity within seven months [79]. In cases where Bankart lesions or glenoid rim fractures were repaired simultaneously, no further episodes of instability were reported [7,19,83].

In a comparative study of 107 patients, Tao et al. evaluated three fixation methods: (1) PHILOS plate (DePuy Synthes, Raynham, MA, USA) via deltopectoral approach, (2) PHILOS plate via deltoid-splitting approach, and (3) a novel anatomical plate via deltoid-splitting approach. All groups were comparable for demographic and injury-related factors. However, patients in the last group experienced less postoperative pain on days three and five [86].

Although most fixation techniques are designed for adult patients, select methods have been applied in pediatric cases. Godin et al. [82] described a double-row arthroscopic suture anchor technique, though no long-term or comparative data with open techniques are currently available. Chillemi et al. [87] reported a transosseous fixation method but cautioned against its use in children with open physes.

A case by Pushpasekaran [88] described fixation of a GTF in a 14-year-old using an extraosseous technique through a deltopectoral approach that preserved the physis. The procedure was initially attempted arthroscopically but converted to open due to concern for physeal damage from multiple anchors. Open surgery offers improved access to the retracted supraspinatus tendon, although the current literature does not clarify whether open procedures are superior to arthroscopy in the pediatric population.

## 8. Limits of the Study

This narrative review is subject to several limitations. Firstly, the literature search did not adhere to a formal systematic review protocol, and no standardized inclusion or exclusion criteria were applied. Consequently, there is potential for selection bias in the studies discussed. Secondly, due to the heterogeneity of the study designs, patient populations, surgical techniques, and outcome measures, it was not feasible to perform a quantitative synthesis or meta-analysis. Furthermore, many of the included studies combined outcomes from isolated GTF and GTFDs, which may confound interpretation, as these entities differ in pathophysiology, treatment considerations, and prognosis.

Additionally, comparative studies directly assessing the outcomes of different surgical techniques for GTFDs remain limited, making it difficult to draw evidence-based conclusions on optimal surgical approaches.

Lastly, pediatric and geriatric populations are underrepresented in the current evidence base, thus limiting the applicability of findings to these specific patient populations.

## 9. Conclusions

GTFDs in the proximal humerus are distinct from isolated fractures in terms of assessment and treatment. Iatrogenic fractures of the humeral neck may occur during glenohumeral reduction, particularly in the presence of unrecognized pre-existing fractures or inadequate muscle relaxation. Minimally displaced or undisplaced GT fragments may be managed conservatively with careful radiographic follow-up to detect secondary displacement. Surgical intervention is recommended for displacements ≥ 5 mm, though thresholds of 3 mm—or even 2 mm in overhead athletes—are increasingly adopted in young, active patients.

A tiered treatment algorithm is proposed: arthroscopic fixation for small or comminuted avulsion-type fragments, open reduction with screws or locking plates for larger split-type fractures with good bone quality, and RSA for elderly patients with poor bone stock or severe comminution. In pediatric cases, open physeal-sparing techniques are preferred; the role of arthroscopy remains unclear.

Further prospective and pediatric-specific studies are needed to validate these stratified approaches and refine clinical decision making.

## Figures and Tables

**Table 1:** Summary of proximal humerus fracture classifications applicable to greater tuberosity involvement.

Description	Types/Criteria	Classification System
Consists of four major groupings based on the number of displaced parts.	- Group 1: humeral head; - Group 2: greater tubercle; - Group 3: lesser tubercle; - Group 4: humeral shaft.	Neer [27]
According to the location of the fracture lines, the existence of articular involvement, and the presence of collapse, deviation, or fragmentation of the metaphyseal region.	- Simple Greater Tuberosity Fracture Extraarticular, Unifocal; - Multifragmentary Greater Tuberosity Fracture—Extraarticular, Wedge, or Comminuted; - Greater Tuberosity Fracture with Articular Involvement—Partial Articular, Shear Fracture.	AO/OTA [30]
Used to classify isolated greater tuberosity fractures and has important therapeutic implications. It is considered reliable for isolated greater tuberosity fractures but is less reliable in multi-part proximal humeral fractures.	- Type 1: avulsion fracture involves a small fragment of bone, and the fracture line is horizontal; - Type 2: split fracture involves a large fragment with a vertical fracture line; - Type 3: depressed fracture involves a fragment that is displaced inferiorly; the fragments are impacted into the humeral head and are generally nondisplaced.	Mutch [18]

## Data Availability

Not applicable.

## Abbreviations

GTFDs	Greater tuberosity fracture–dislocations
ORIF	Open reduction and internal fixation
RSA	Reverse shoulder arthroplasty
AP	Anteroposterior
CT	Computed Tomography
MRI	Magnetic Resonance Imaging
TENS	Transcutaneous electrical nerve stimulation
ROM	Range of motion
AO/OTA	Arbeitsgemeinschaft für Osteosynthesefragen/Orthopedic Trauma Association
